# Anodized Nanostructured 316L Stainless Steel Enhances Osteoblast Functions and Exhibits Anti-Fouling Properties

**DOI:** 10.1021/acsbiomaterials.2c01072

**Published:** 2023-01-24

**Authors:** Yasar Kemal Erdogan, Batur Ercan

**Affiliations:** † Biomedical Engineering Program, Middle East Technical University, Ankara 06800, Turkey; ‡ Department of Biomedical Engineering, Isparta University of Applied Science, Isparta 32260, Turkey; § Department of Metallurgical and Materials Engineering, Middle East Technical University, Ankara 06800, Turkey; ∥ BIOMATEN, METU Center of Excellence in Biomaterials and Tissue Engineering, Ankara 06800, Turkey

**Keywords:** 316L stainless steel, anodization, topography, nanostructure, anti-fouling

## Abstract

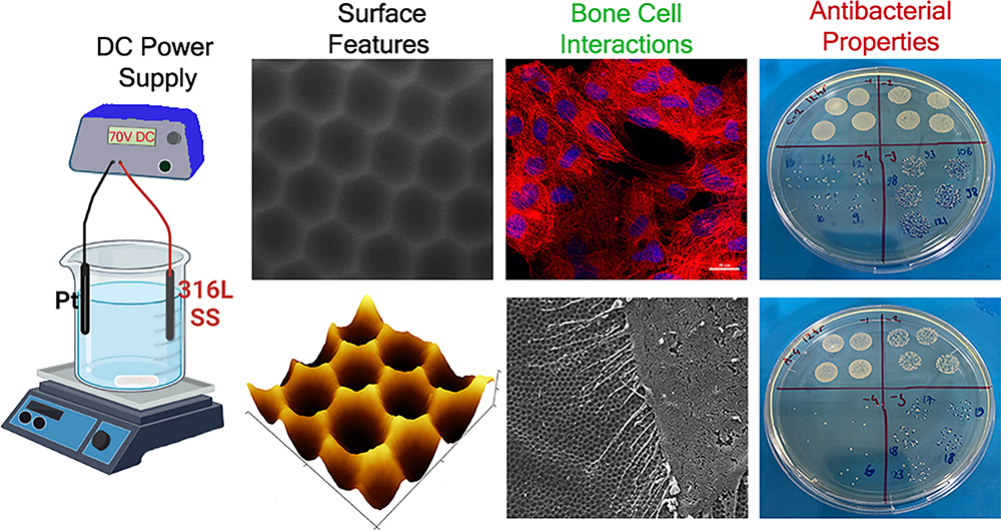

Poor osseointegration and infection are among the major challenges of 316L stainless steel (SS) implants in orthopedic applications. Surface modifications to obtain a nanostructured topography seem to be a promising method to enhance cellular interactions of 316L SS implants. In this study, arrays of nanodimples (NDs) having controlled feature sizes between 25 and 250 nm were obtained on 316L SS surfaces by anodic oxidation (anodization). Results demonstrated that the fabrication of NDs increased the surface area and, at the same time, altered the surface chemistry of 316L SS to provide chromium oxide- and hydroxide-rich surface oxide layers. In vitro experiments showed that ND surfaces promoted up to a 68% higher osteoblast viability on the fifth day of culture. Immunofluorescence images confirmed a well-spread cytoskeleton organization on the ND surfaces. In addition, higher alkaline phosphate activity and calcium mineral synthesis were observed on the ND surfaces compared to non-anodized 316L SS. Furthermore, a 71% reduction in *Staphylococcus aureus* (*S. aureus*) and a 58% reduction in *Pseudomonas aeruginosa* (*P. aeruginosa*) colonies were observed on the ND surfaces having a 200 nm feature size compared to non-anodized surfaces at 24 h of culture. Cumulatively, the results showed that a ND surface topography fabricated on 316L SS via anodization upregulated the osteoblast viability and functions while preventing *S. aureus* and *P. aeruginosa* biofilm synthesis.

## 1 Introduction

316L stainless steel (316L SS) is one of the commonly used metallic implant materials owing to its optimal mechanical properties, superior wear resistance, adequate corrosion resistance, and easy processability.^1^ 316L SS also provides a significant price advantage compared to other metallic biomaterials (i.e., titanium and titanium alloys), which makes it the material of choice to fabricate orthopedic implants in most developing and underdeveloped countries across the world.^2,3^ However, the bioinert nature of 316L SS hinders the desired cellular response; effective new bone formation and osseointegration are limited in orthopedic applications.^4^ Inadequate osseointegration would lead to micromotion of the implant and, in the long run, failure due to aseptic loosening.^5^ Aside from lacking bioactivity, another important problem of 316L SS implants is their septic failure. Since 316L SS does not possess any antibacterial activity, once bacteria attach and subsequently form a biofilm on its surfaces, it is very difficult to eradicate it. Frequently, it requires a revision surgery and replacement of the implant. Considering that most bacteria build resistance to commonly used antibiotics, fighting with infection is becoming a pressing issue.

Creating a nanostructured topography on implant surfaces is a promising strategy to improve the biological response and prevent bacterial colonization on 316L SS implant surfaces.^6,7^ Anodic oxidation (anodization) is an electrochemical surface modification technique to form a nanostructured oxide layer on the surfaces of valve metals, including aluminum,^8,9^ titanium,^10,11^ zirconium,^12,13^ etc. In the last decade, anodization of different metallic implant components received considerable attention due to it is simplicity and ability to obtain nanofeatures having different feature sizes and morphologies by changing the anodization parameters, i.e., voltage, time, etc.^14−17^ For instance, our research group previously created nanopores, nanodimples (NDs), nanotubes, and nanocoral morphologies on tantalum surfaces and controlled the feature size of these morphologies between 20 and 140 nm to enhance the osteoblast (bone cell) functions on these surfaces.^18^ In another study, we formed nanotubular structures on titanium surfaces via anodization and controlled the feature size of these nanotubular structures between 25 and 140 nm. The nanotubular structures improved exosome secretion, which in turn stimulated the endothelial cell viability.^19^ In another study, aluminum was anodized to obtain nanopores having a pore size range of 25–75 nm, and it was found that the larger pore size on anodized aluminum dramatically enhanced cellular proliferation.^20^

Several studies investigated the fabrication of nanostructures on 316L SS surfaces via anodization and its effects on surface properties,^21,22^ cellular functions,^23,24^ and bacterial colonization.^25,26^ For example, anodized 316L SS surfaces containing both micro- and nanopores were shown to induce osteoblast-like cell adhesion, which was correlated with increased surface roughness.^27^ In a recent study, anodized 316L SS implants were shown to improve bone recovery 4 weeks after implantation and supported osseointegration. However, these anodized implants had non-uniform and unordered nanostructures on implant surfaces.^28^ Aside from tissue interactions, various studies identified the nanostructured surface topography to inhibit attachment and growth of bacteria.^29,30^ Though the anodization method was not used, nanoscale surface topography on SS was shown to be critical in limiting *Staphylococcus aureus* (*S. aureus*) and *Pseudomonas aeruginosa* (*P. aeruginosa*) colony formation.^31^ Jang et al. showed that nanotextured 316L SS surfaces fabricated by electrochemical etching significantly inhibited *Escherichia coli* (*E. coli*) and *S. aureus* attachment. Though nanofeatures were identified to inhibit bacteria colonization, it should be noted that aforementioned nanotextures were irregular and did not possess an array of repeating nanostructured topographical features.^32^

Although nanofeatured surfaces on 316L SS were fabricated via different techniques to interact with various tissues, most of the fabricated surface features were non-uniform, could not be scaled-up for large curved areas, and were prone to delamination failure, which would limit their use in orthopedic applications. Anodization of 316L SS to provide uniform and controllable nanofeatures would be advantageous, and optimization of the nanofeature size to enhance bone cell functions and anti-fouling properties is required for their adaptation in orthopedic applications. Therefore, the aim of the present study is to identify a surface topography that enhances bone cell interactions and, at the same time, limit bacterial colonization on 316L SS. For this purpose, we fabricated and characterized ND structures on 316L SS using anodization. The effect of the ND size on bone cell functions and anti-fouling properties against Gram-positive *S. aureus* and Gram-negative *P. aeruginosa* was investigated.

## 2 Materials and Methods

### 2.1 Sample Preparation

An austenitic 316L stainless SS foil (0.5 mm) was cut into 1 × 1 cm-sized samples. Prior to anodization, the samples were ultrasonically cleaned in acetone, ethanol, and distilled water each for 10 min. For anodization, the samples were connected to a DC power supply (Genesys 300V/5, TDK Lambda), which had a two-electrode configuration. A platinum mesh was used as the cathode and a 316L SS sample was used as the anode. 316L SS samples were anodized in ethylene glycol monobutyl ether (EG, Sigma-Aldrich) solution containing 7.5% (v/v) perchloric acid (HClO_4_, Sigma-Aldrich) to obtain nanofeatured surfaces. Anodization experiments were carried out at temperatures lower than 6 °C. To control the feature size of the nanofeatures, the applied potentials were altered between 25 and 80 V, and the anodization durations were set between 1 and 20 min. After the anodization process, all the samples were rinsed with distilled water and dried at room temperature.

### 2.2 Surface Characterization

The surface morphology of the non-anodized (NA) and anodized 316L SS surfaces were investigated using a scanning electron microscope (SEM, FEI Nova Nano 430) equipped with a secondary electron detector. For analysis of the nanofeature dimensions, the measurements were completed from 30 different surface features in triplicate using ImageJ 1.51 software (National Institute of Health). An atomic force microscope (AFM, Veeco, Multimode V) was used to characterize the nanoscale roughness of the samples. Surfaces were scanned in tapping mode using a silicon AFM tip having a 10 nm radius. For each sample, 1 × 1 μm^2^ fields were analyzed at a rate of 1 Hz. The AFM data were analyzed using Image Plus software. The micron-scale roughness values of the samples were measured using a profilometer (MarSurf PS 10) from at least three different locations. The hydrophobicity of the samples was characterized using a goniometer (EasyDrop, KRÜSS GmbH). Ultrapure water (8 μL) was dropped onto each sample, and the sessile drop water contact angles at the sample interface were measured. The chemical composition of the outermost surface oxide layer on 316L SS was characterized using an X-ray photoelectron spectroscope (PHI 5000 Versa Probe) equipped with a monochromatic Al Kα X-ray source. High-resolution spectra of the Cr 2p, Fe 2p, O 1s, and C 1s peaks were obtained. The C 1s peak was used as the reference and set at 284.8 eV. Curve fitting of the peaks was performed with XPSPeak 41 software.

### 2.3 Cytotoxicity Testing

Human osteoblasts (hFOB 1.19, ATCC CRL-11372) were cultured using Dulbecco’s modified Eagle’s medium (DMEM, Sigma-Aldrich) supplemented with 10% fetal bovine serum (FBS, Sigma-Aldrich), 1% penicillin–streptomycin (Sigma-Aldrich), and 1% l-glutamine (Sigma-Aldrich) under standard cell culture conditions (37 °C and 5% CO_2_). Prior to cell culture, 316L SS samples were sterilized with 70% ethanol for 15 min, followed by UV sterilization for 30 min. 3-[4,5-Dimethylthiazol-2-yl]-2,5-diphenyl tetrazolium bromide (MTT) assay was used to assess the cell–surface interactions. hFOBs were seeded onto sterile 316L SS samples at a density of 10^4^ cells/cm^2^, and the cells were cultured up to 5 days in vitro under standard cell culture conditions (37 °C and 5% CO_2_). After the prescribed time periods, the samples were rinsed with 1× PBS and transferred to fresh wells. 500 μL of MTT solution (1 mg/mL) was added onto each sample, and the samples were incubated for 4 h to form formazan crystals. The formazan product was solubilized in 0.1 M HCL solution prepared in isopropanol. 250 μL of the dissolved solution from each sample was transferred to obtain absorbance readings at 570 using a microplate reader (Thermo Scientific Multiskan Go). The absorbance values of the samples without cells (blank) were subtracted from the obtained absorbance data. MTT experiments were repeated three times with three samples in each replicate.

### 2.4 Cellular Imaging

The cellular morphology was assessed both with SEM and immunofluorescence imaging. Prior to imaging, hFOBs seeded onto sterile samples were cultured under standard cell culture conditions (37 °C and 5% CO_2_). On the third day of culture, hFOBs were fixed using 4% paraformaldehyde (Sigma-Aldrich) for 20 min. For SEM imaging, the fixed cells were dehydrated with 30, 70, 90, 95, and 100% (v/v) ethanol for 10 min each. Afterward, the samples were treated with hexamethyldisilazane (Sigma-Aldrich) and left to dry for 12 h. The dried samples were coated with gold using a sputter coater, and the images were captured using a scanning electron microscope (FEI Nova Nano 430). For immunofluorescence imaging, the fixed cells were permeabilized with 0.2% Triton X-100 for 30 min and blocked with 5% BSA for 30 min. Actin fibers were stained with phalloidin prepared at a 1:200 dilution factor (Abcam) for 1 h. The nuclei of the cells were stained with a 4′,6-diamidino-2-phenylindole dihydrochloride (DAPI) solution prepared at a 1:40000 dilution for 30 min. Images were captured using a confocal microscope (Zeiss LSM800) and merged with ZEISS ZEN Imaging Software. Quantitative analysis was completed using ImageJ (NIH) in triplicate using three replicates for each experiment.

### 2.5 Cellular Functions

To assess the hFOB functions, the cells were incubated for up to 5 weeks using an osteogenic cell medium (DMEM F-12, 10% FBS, 1% l-glutamine, 1% penicillin–streptomycin, 50 μg/mL l-ascorbic acid, 0.01 μM dexamethasone, and 10 mM β-glycerophosphate) under standard cell culture conditions (37 °C and 5% CO_2_). To measure the alkaline phosphatase activity (ALP) activity, hFOBs were seeded at a density of 2 × 10^4^ cells/cm^2^ onto sterile samples. At 2, 3, 4, and 5 weeks of culture, the samples were rinsed with 1× PBS and transferred to fresh wells, followed by the addition of 400 μL of 0.2% Triton X solution for 5 min to lyse the cells. The cell lysates were centrifuged at 14000 rpm for 5 min at 4 °C to collect supernatants. ALP activities were assessed using a commercially available kit (ab83369, Abcam) following the manufacturer’s instructions. Absorbance values were obtained using a microplate reader (Thermo Scientific Multiskan Go) at 405 nm. The ALP activity of hFOBs were determined using a standard absorbance–concentration curve of phosphate run in parallel. For alizarin red staining, 3 × 10^4^ cells/cm^2^ were seeded onto sterile samples. At 2, 3, 4, and 5 weeks of culture, the cells were fixed with 4% paraformaldehyde for 20 min. After rinsing the samples with distilled water, 500 μL of a 40 mM alizarin red solution (pH = 4.2) was added onto each sample, and the samples were kept for 30 min at 80 rpm. Then, each sample was rinsed with distilled water once more to remove unbonded alizarin red. Calcium deposited on the samples were visualized using an optical microscope (Huvitz HDS-5800). For quantitative analysis, 500 μL of a 10% cetylpyridinium chloride solution prepared in 10 mM disodium phosphate was added onto each sample, followed by mixing the samples in the dark for 1 h at 80 rpm. At the end of 1 h, the absorbance values were measured using a microplate reader (Thermo Scientific Multiskan) at 562 nm. ALP and alizarin red assays were performed in triplicate using three replicates for each experiment.

### 2.6 Anti-Fouling Performance

Bacterial tests were conducted with Gram-positive *S. aureus* (*S. aureus*, ATCC 25923) and Gram-negative *P. aeruginosa* (*P. aeruginosa*, ATCC 27853) to evaluate the anti-fouling properties of the samples. Bacteria were taken from the stock culture and streaked onto a tryptic soy broth (TSB) agar plate. After 24 h, a single colony from the agar plate was inoculated into 3% TSB and incubated at 37 °C for 18 h at 200 rpm. *S. aureus* and *P. aeruginosa* bacterial suspensions were prepared according to 0.5 McFarland standards and then further diluted to 1:100 with 1× PBS prior to seeding. 0.5 mL of bacteria solution was added onto each sample, and bacteria were allowed to adhere for 1 h. After 1 h, the samples were gently rinsed with 1× PBS, transferred to fresh wells, and incubated with 0.3% TSB at 37 °C up to 24 h. At 1, 4, 12, and 24 h of culture, the samples were transferred to sterile tubes containing 1× PBS. Each sample was vortexed for 90 s to detach the bacteria from the sample surfaces into the PBS solution. Afterwards, PBS solution containing the bacteria was serially diluted, followed by plating 20 μL of each dilution onto agar plates. After 18 h of incubation, the number of colonies on the agar plates was counted and reported per milliliter of bacteria solution for each sample. To visualize the adherent bacteria, samples inoculated with *S. aureus* or *P. aeruginosa* were gently rinsed with 1× PBS at 4 and 24 h of culture. The SEM imaging protocol detailed in Section 2.4 was followed.

Biofilm formation on the samples was evaluated by crystal violet (CV) staining. Similar bacteria seeding and culture protocol were followed. After 24, 48, and 72 h of incubation, media on the samples were aspirated. Afterward, the samples were washed with 1× PBS and transferred to fresh wells. The biofilm was stained with 500 μL of 0.1% CV solution for 15 min. Once staining was completed, CV solutions were aspirated, and the samples were gently washed three times with 1× PBS. Once the samples were dry, 500 μL of ethanol (99%) solution was added for 15 min to dissolve the CV dye staining the biofilm. Optical density values were measured at 562 nm.

### 2.7 Statistical Analysis

SPSS software was used for the statistical analysis. One-way analysis of variance with Tukey’s test was performed for data analysis. *p* < 0.05 was considered statistically significant.

## 3 Results and Discussion

### 3.1 Surface Characterization

**Figure 1:**
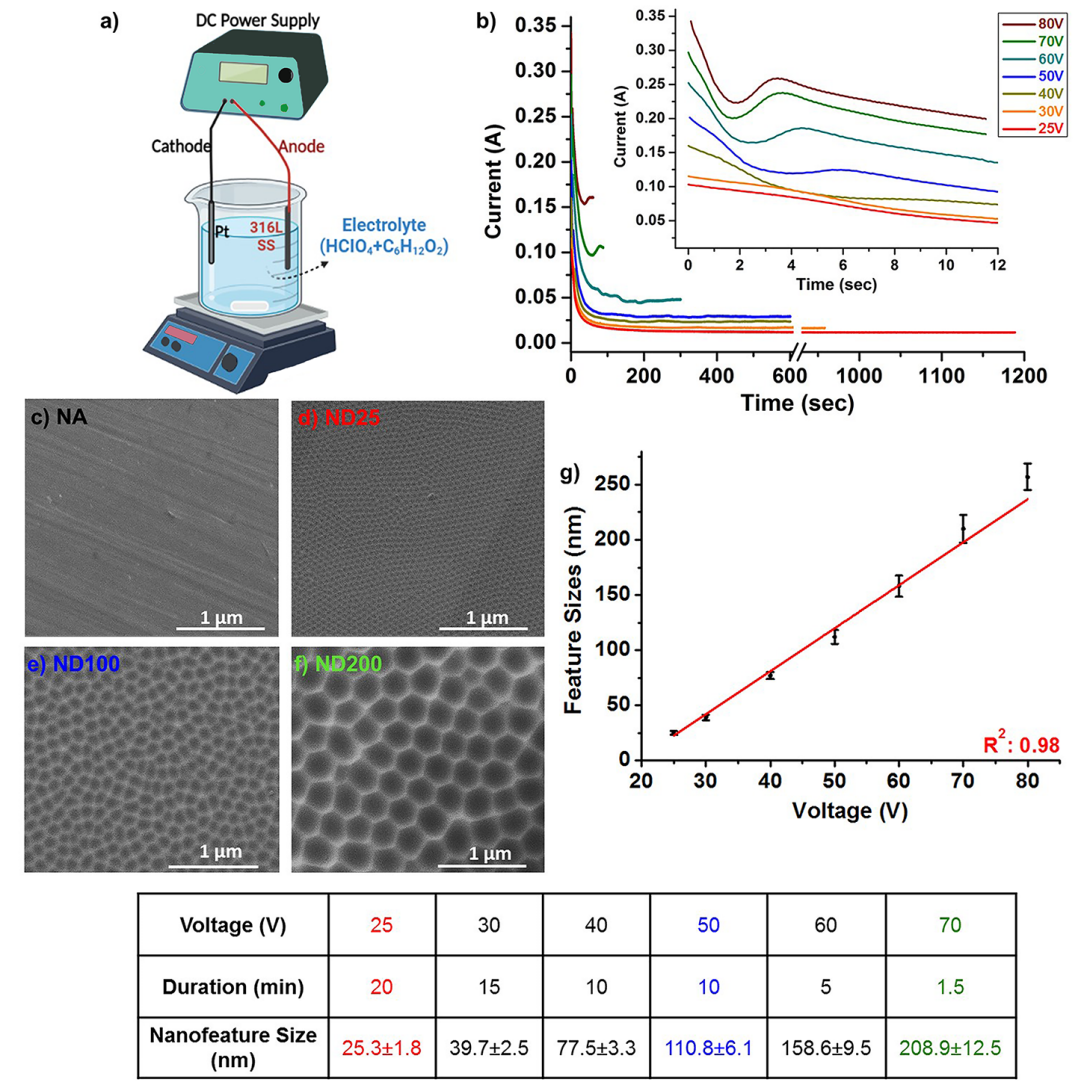
(a) Schematic of the anodization set-up. (b) Current–time graphs obtained during anodization of 316L SS. SEM micrographs of (c) NA, (d) ND25, (e) ND100, and (f) ND200. (g) ND feature size vs voltage graph for anodized 316L SS. Feature size of NDs increased with an increase in the applied voltage during anodization. The table shows the anodization voltage, duration, and obtained nanofeature size.

Figure 1a shows the schematic of the electrochemical set-up used for the anodization experiments, where a platinum mesh was used as the cathode and 316L SS samples were the anode. The current–time (*I*–*t*) graphs obtained during anodization of the 316L SS samples are shown in Figure 1b. As evident in the *I*–*t* curves, the formation of nanostructured surfaces consisted of three characteristic regions when higher voltages were used. In the first region, the current passing through the system decreased swiftly, which was a result of oxide layer formation on the 316L SS surfaces. This was followed by a second region where current passing through the system increased. In the second region, dissolution of the oxide layer started and formation of nanostructures began. Finally, gradual decrease in the current was observed in the third region due to the limited ionic diffusion across the oxide layer on the surfaces. The formation of three distinct regions on the *I*–*t* graph was in line with the literature for anodization of SS samples.^33,34^ In contrast, these three characteristic regions in the *I*–*t* graph did not appear when lower voltages were used during anodization. This was primarily due to lower voltages not being able to provide enough of a driving force for voltage-induced oxide layer formation and dissolution mechanisms to dominate over each other at specific regions of the *I*–*t* graph. Though oxide layer formation and dissolution mechanisms were still active, three distinct regions were not evident on the *I*–*t* graph. After anodization of 316L SS, the surface morphology was verified with SEM (Figure 1c–f). It was observed that highly ordered, uniform nanostructures formed on the 316L surfaces. The obtained nanostructures had a dimple-like morphology, and thus these anodized samples were referred as ND samples. It was observed that the ND size was sensitive to the anodization voltage;^22^ as the applied voltage was increased, the feature size of NDs increased, as well. In fact, arrays of NDs having a controlled and uniform feature size ranging from 25 to 250 nm were fabricated by altering the applied voltage (Figure 1c–f). Figure 1g shows that there was a linear relationship between the applied voltage and the feature size of the NDs forming on 316L SS (*R*^2^: 0.98). In this study, we selected three different ND sizes for sample characterization to cover both nano- and submicron-sized regimes. The selected ND sizes were 25.3 ± 1.8, 110.8 ± 6.1, and 208.9 ± 12.5 nm, and these samples were referred to as ND25, ND100, and ND200, respectively. In addition, the NA 316L SS samples were used as control, and they were referred to as NA.

**Figure 2:**
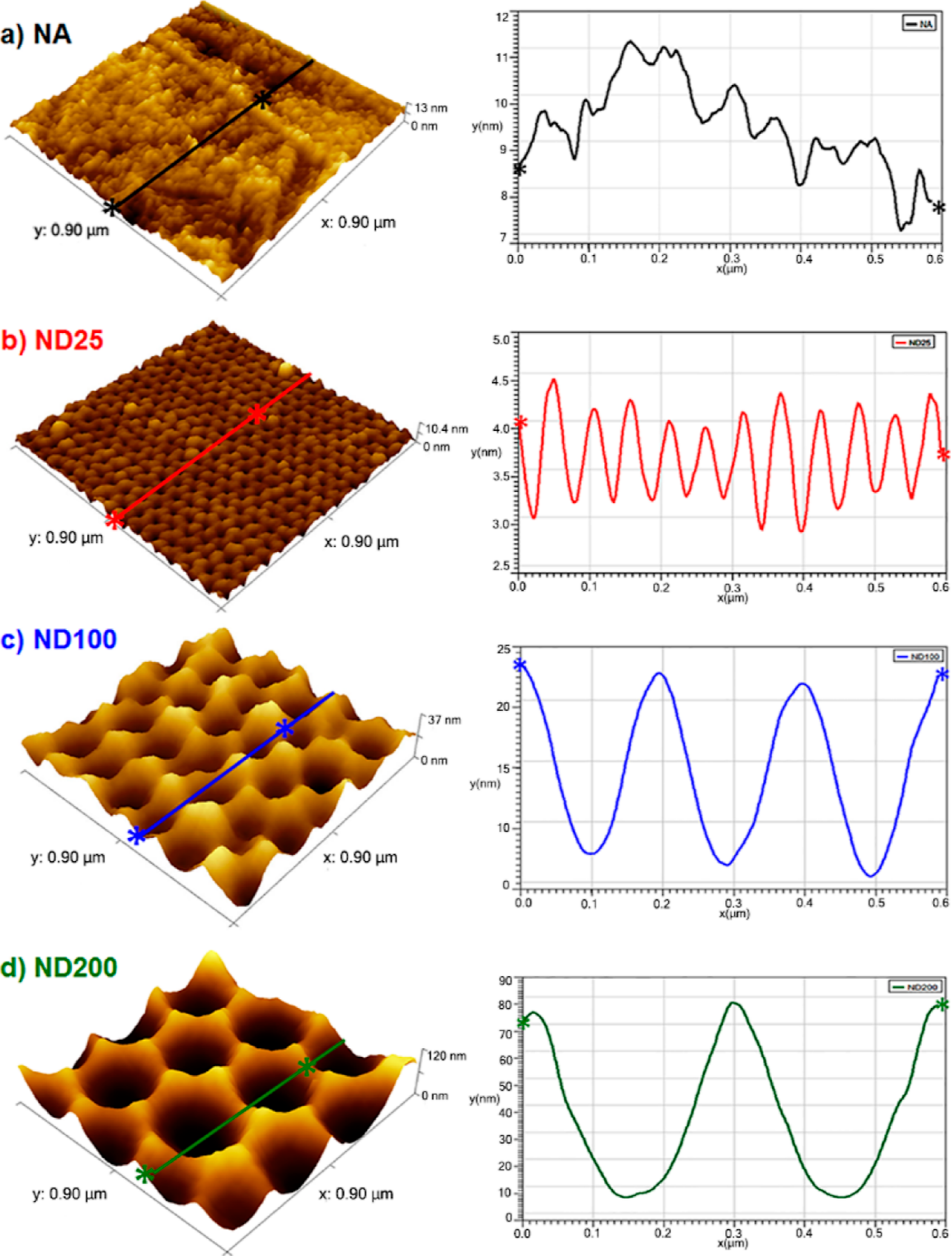
AFM images and the corresponding roughness profiles obtained across the highlighted lines for (a) NA, (b) ND25, (c) ND100, and (d) ND200.

Figure 2 shows the AFM micrographs and the corresponding roughness profiles across the given lines on the sample surfaces. These figures verified that the anodization process created a unique nanostructured topography on 316L SS surfaces. The root mean square roughness values (Sq) calculated from the AFM scans were 1.5 ± 0.2, 2.9 ± 0.5, 9.7 ± 1.2, and 20.1 ± 2.1 nm for the NA, ND25, ND100, and ND200 samples, respectively (Table 1). Furthermore, the surface areas of the samples were calculated to be 0.94 ± 0.09, 0.97 ± 0.11, 1.10 ± 0.14, and 1.74 ± 0.27 μm^2^ for the NA, ND-25, ND-100, and ND-200, respectively (Table 1). It was clear that the nanoscale surface roughness and surface area of 316L SS increased upon anodization. In fact, the increase in ND feature size increased both the surface roughness and surface area. The ND200 sample had the highest nanoscale roughness (*p* < 0.05) and had the largest surface area (*p* < 0.01) compared to that of the NA. It should be noted that the surface roughness measurements and area calculations were limited with the resolution of the AFM tip to interact with the dimple-shaped nanostructures on the 316L SS surfaces. Thus, surface morphologies could only be partially represented on the AFM micrographs. In addition, the roughness profile of the ND structures increased with an increase in ND feature size, which were measured to be around 1 ± 0.3, 16 ± 1.8, and 70 ± 2.6 nm for ND25, ND100, and ND200 surfaces, respectively. However, when the micron scale surface roughness was measured, there was no significant difference between ND and NA surfaces (Table 1). These results indicated that the anodization process did not change the micrometer surface roughness, yet increased the nanoscale roughness of the surfaces.

**Table 1:** Nano- and Micron-Scale Surface Roughness Values, Surface Areas, and Sessile Drop Water Contact Angles for NA, ND25, ND100, and ND200 Samples, *p < 0.05 and **p < 0.01

sample	nanoscale roughness (Sq, nm)	micron scale roughness (Rq, μm)	surface area (μm^2^)	water contact angle (°)
NA	1.5 ± 0.2	0.13 ± 0.01	0.94 ± 0.09	63.6 ± 1.9
ND25	2.9 ± 0.5	0.12 ± 0.01	0.97 ± 0.11	69.7 ± 3.5
ND100	9.7 ± 1.2*	0.13 ± 0.01	1.10 ± 0.14	65.3 ± 3.8
ND200	20.1 ± 2.1**	0.13 ± 0.01	1.74 ± 0.27*	72.6 ± 3.6*

Sessile drop water contact angle values for the samples are given in Table 1. All the samples investigated in this study showed hydrophilic characteristics independent of having NDs or not. ND200 samples had a slightly higher water contact angle compared to that of NA (*p* < 0.05). This result could be explained with the Cassie–Baxter theory, which included the effect of surface roughness on the contact angle. Since the anodization process created NDs on 316L SS surfaces, it was possible that water did not completely penetrate through the NDs and left some air gaps, which led to an increase in the water contact angle value of ND200.

**Figure 3:**
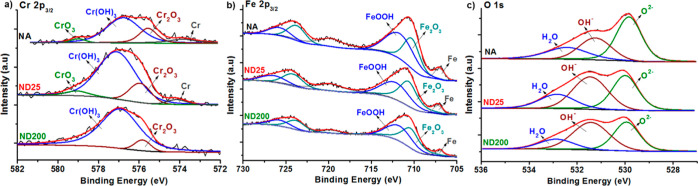
High-resolution XPS spectra of NA, ND25, and ND200 samples obtained for (a) Cr 2p_3/2_, (b) Fe 2p_3/2_, and (c) O 1s peaks.

High-resolution XPS spectra of NA, ND25, and ND200 samples are provided in Figure 3. Curve fitting for chromium peaks (Figure 3a) showed that NA surfaces consisted of Cr(0) (metallic chromium), Cr_2_O_3_, Cr(OH)_3_ (hydrated chromium oxide), and CrO_3_ components and expressed peaks at around 573.8, 575.5, 576.8, and 579.0 eV, respectively.^34^ Upon anodization, Cr(0), Cr_2_O_3_, Cr(OH)_3_, and CrO_3_ peaks for the ND25 samples slightly shifted to 574.1, 575.9, 577.1, and 579.0 eV, respectively. For ND200 surfaces, Cr_2_O_3_, Cr(OH)_3_, and CrO_3_ peaks shifted to 575.8, 576.9, and 578.9 eV, respectively.^23^ The anodization process altered the chemical composition of the outermost layer of 316L SS and led to an increase in the Cr(OH)_3_ content for ND25 and ND200 surfaces compared to NA. Changes in the oxidation state of chromium led to higher binding energies in the Cr 2p spectra. Metallic Cr on the surfaces was oxidized during anodization. However, the Cr(0) peak was still apparent for the ND25 surfaces due to lower potentials used during anodization. On the other hand, the Cr(0) peak disappeared from the XPS spectrum for the ND200 surfaces, which could be correlated with accelerated oxidation at higher voltages. Similarly, the anodization process affected the chemical composition of Fe 2p at the 316L SS surfaces. Figure 3b shows the curve fitting for Fe 2p_3/2_ peaks, where NA surfaces consisted of metallic iron (Fe(0)), Fe_2_O_3_, and FeOOH at 707.0, 710.2, and 711.7 eV, respectively.^35^ Upon the anodization of 316L SS, Fe(0), Fe_2_O_3_, and FeOOH peaks for the ND25 surfaces slightly shifted to 707.2, 710.6, and 712.2 eV, respectively. For the ND200 surfaces, Fe(0), Fe_2_O_3_, and FeOOH peaks appeared at 707.0, 710.4, and 712.1 eV, respectively.^36^ The intensity of the Fe(0) peak decreased on the anodized surfaces compared to the NA surfaces. When peak intensities were compared, the lowest Fe(0) intensity was observed for the ND200 surfaces, followed by the ND25 surfaces, and the highest Fe(0) intensity was observed for the NA surfaces. On the other hand, an opposite trend was observed for Fe_2_O_3_, where the highest Fe_2_O_3_ intensity was observed for the ND200 surfaces, followed by ND25 surfaces, and the lowest peak intensity was observed for the NA surfaces. This result was an indication for oxidation of the metallic Fe and formation of Fe_2_O_3_ on the surfaces of 316L SS upon anodization. When the XPS peaks were carefully investigated, it was observed that anodization reaction led to slight shifts for all of the chromium and iron peaks toward higher binding energies. This could be attributed to the diffusion of oxygen anions into the 316L SS matrix during the anodization process. When negatively charged oxygen anions migrate toward the positively charged 316L SS anode, their concentration inside the 316L SS lattice increased, which led to higher binding energies for the electrons. In the literature, the color change of the electrolyte used during anodization was provided as an indirect validation of Fe-oxide layer dissolution during anodization,^37^ and our findings were in line with this assessment. When Cr(oxide + hydroxide)/Fe(oxide + hydroxide) ratios of the surfaces were analyzed, NA surfaces had a ratio of 0.20, while this ratio was 0.44 and 0.48 for ND25 and ND200 surfaces, respectively. This result indicated the formation of chromium oxide and hydroxide, while iron oxide and hydroxide contents diminished at the ND surface layers. The high-resolution spectrum of O 1s (Figure 3c) was curve-fitted with three different peaks at 529.8, 531.2, and 532.4 eV for the NA surfaces, and these peaks were assigned to metal-oxides, hydroxides, and adsorbed water, respectively.^23,36,38^ These peaks on ND25 and ND200 surfaces shifted to 529.9, 531.4, and 532.8 eV. The intensity of the hydroxide peak significantly increased on ND25 and ND200 compared to NA. The hydroxylation degree (OH^–^/O^2–^) was quantitatively analyzed to be 0.91, 1.09, and 1.28 for NA, ND25, and ND200 surfaces, respectively. The overall increase in the OH^–^ content might provide some negative charge for the ND25 and ND200 surfaces.^39^ The increased intensity of the Cr(OH)_3_ peak (Figure 3a) supported the formation of hydroxyl groups on the oxide layer (Figure 3c). This was related to oxidation and dissolution of Cr under the applied voltages.^6^ When a voltage was applied, initially, chromium-oxide formed on the 316L SS surfaces. Afterward, this layer dissolved inside the electrolyte to form chromium-hydroxide, whose XPS peak was observed at higher binding energies.^40^ Overall, XPS results showed that anodization process changed the surface chemical layer composition, which might have contributed to the effects on the biological and antibacterial properties of 316L SS.

### 3.2 Osteoblast Interactions

**Figure 4:**
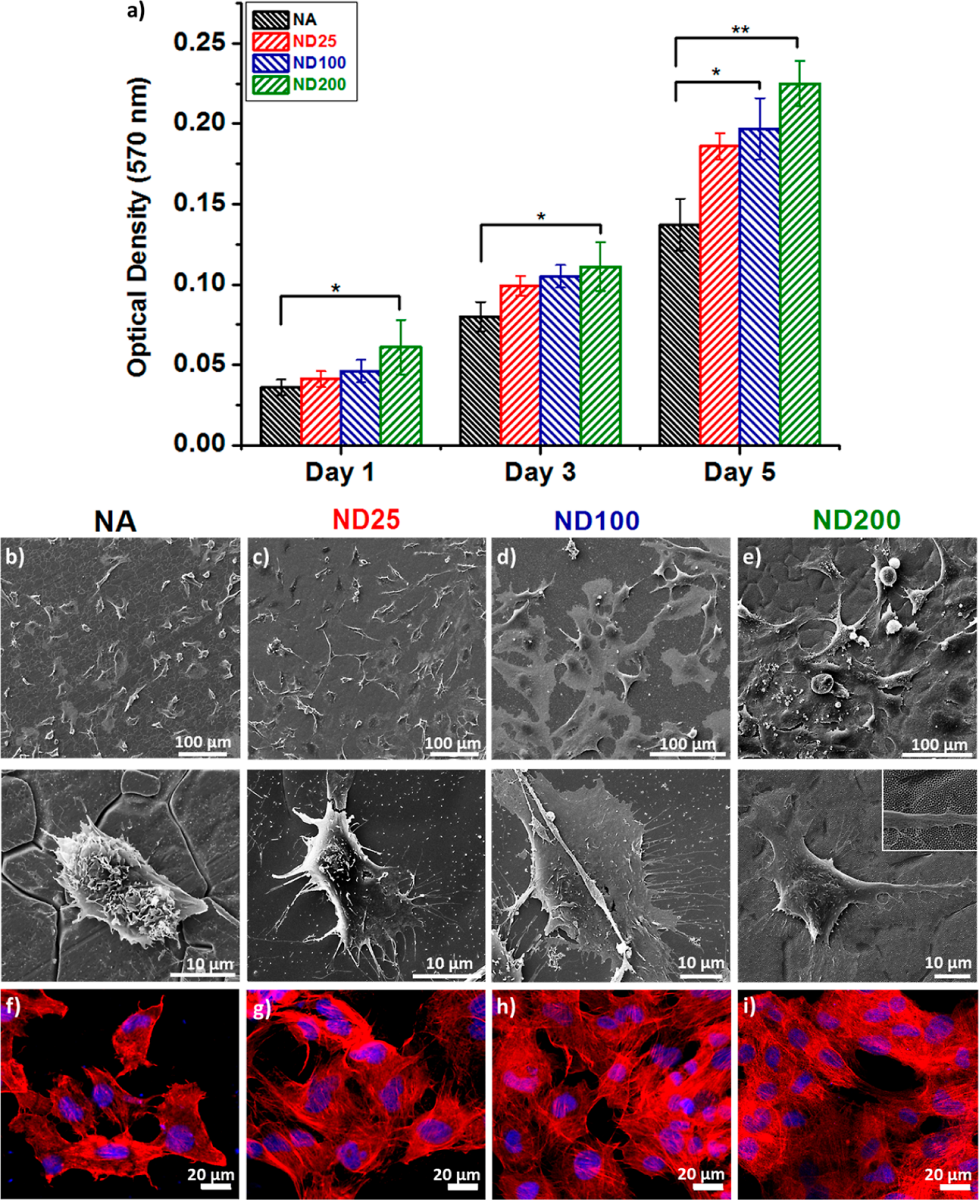
(a) Osteoblast viability on NA, ND25, ND100, and ND200 up to 5 days in vitro (*p < 0.05, and **p < 0.01). (b–e) SEM micrographs. (f–i) Nuclei (blue) and f-actin (red)-stained osteoblasts on (b,f) NA, (c,g) ND25, (d,h) ND100, and (e,i) ND200 on the third day of culture.

Figure 4 shows that osteoblasts were viable and successfully proliferated on NA, ND25, ND100, and ND200 surfaces up to 5 days in vitro. ND200 significantly enhanced hFOB proliferation compared to NA on the first and third days of culture (*p* < 0.05). At the end of the fifth day, ND200 and ND100 both promoted higher hFOB viability compared to NA (**p < 0.01 and *p < 0.05). The reason for the increased cellular viability on ND200 could be explained with its unique surface properties. Specifically, ND200 had a higher nanoscale roughness and surface area compared to NA (Table 1). The increased surface area promoted higher osteoblast adhesion, proliferation, and viability on ND200. Similarly, ND-like structures on anodized 304L SS promoted osteoblast-like cell viability and functions. However, the aforementioned study did not investigate features less than a 100 nm pore size.^24^ Similarly, Beltrán-Partida et al. reported that the anodization process increased the surface roughness, resulting in enhanced cellular viability on nanostructured titanium surfaces.^41^ SEM micrographs of osteoblasts after 3 days of culture are presented in Figure 4b–e. These micrographs demonstrated that hFOB interacted to a greater extent with the ND samples compared to the NA sample. SEM images clearly showed hFOBs expressing more filopodia on ND surfaces compared to NA. The cellular morphology and cytoskeletal organization were also investigated with fluorescence staining (Figure 4f–i), where the blue dye stained the nuclei and the red dye stained the f-actin filaments. Fluorescent microscopy images revealed a well-spread cellular morphology with well-organized f-actin fibers for hFOBs on ND surfaces. Quantitative analysis of hFOB revealed that cells covered a surface area of 1.3 ± 0.1, 2.2 ± 0.3*, 2.1 ± 0.3*, and 2.2 ± 0.2* (10^–3^ × mm^2^/cell) for NA, ND-25, ND-100, and ND-200 samples, respectively. Clearly, osteoblasts spread more on ND samples compared to NA (*p < 0.05). The differences in cell–surface interactions could be attributed to the changes in the surface topography of the ND samples.^42^ It has been suggested in the literature that cellular adhesion was sensitive to nanoscale roughness of the surfaces, which led to enhanced cellular adhesion and proliferation.^27,28^ The existence of numerous filopodia extensions and longer actin filaments on ND surfaces indicated that nanoscale topography provided a suitable environment for cell–surface interactions.

**Figure 5:**
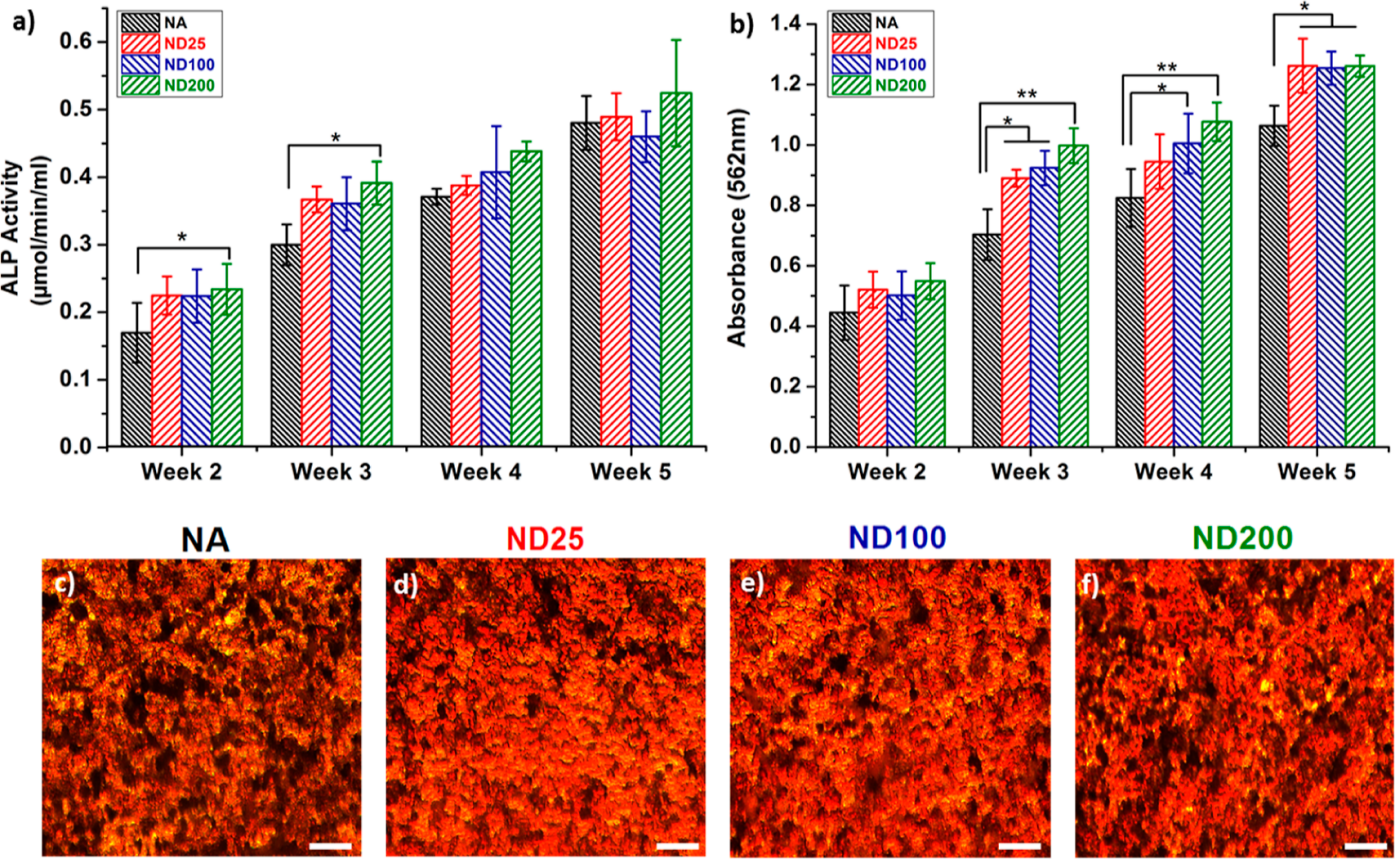
(a) Alkaline phosphate activity and (b) calcium mineral deposition of osteoblasts cultured on NA, ND25, ND100, and ND200 for up to 5 weeks (*p < 0.05, and **p < 0.01). Alizarin red-stained images of (c) NA, (d) ND25, (e) ND100, and (f) ND200 surfaces showing deposited calcium minerals at the third week of culture. Scale bars are 50 μm.

To investigate hFOB functions on NA, ND25, ND100, and ND200 surfaces, ALP and calcium mineral deposition assays were completed for up to 5 weeks in vitro (Figure 5). It was observed that at the second and third weeks of culture, hFOBs cultured on ND200 expressed a higher ALP activity compared to NA (*p* < 0.05). However, at the fourth and fifth weeks of culture, no difference was observed between the sample groups (Figure 5a). ALP played an important role in early bone formation, and it was an early stage marker to indicate osteogenic differentiation of osteoblasts. This was evident in our results where nanostructured surfaces promoted higher ALP activity at early time points as it was required for successful bone synthesis in vivo. To assess calcium mineral deposition of hFOBs, alizarin red staining was conducted for NA, ND25, ND100, and ND200 surfaces for up to 5 weeks in vitro (Figure 5b). It was observed that mineralization gradually increased with the cell culture time after the second week in vitro. NA samples consistently had the lowest mineralization at each time point. At the third and fourth weeks of culture, ND200 expressed the highest mineralization compared to NA surfaces (*p* < 0.01). At the fifth week of culture, all ND surfaces promoted higher calcium mineral deposition compared to NA (*p* < 0.05). The optical microscope images of the alizarin red-stained samples demonstrated abundant calcium deposits on ND surfaces at the third week in vitro. These images qualitatively confirmed absorbance readings and confirmed higher mineral content (red color) on the ND surfaces compared to that of NA. Since cellular spreading was shown to promote cellular functions, enhanced ALP activity and calcium mineral synthesis on ND surfaces could be attributed to the well-spread hFOB morphology on the ND surfaces.^42^

The nanoscale topography of the surfaces significantly affects the adsorption of the RGD peptide-bearing proteins, vitronectin, fibronectin, laminin, and collagen, which stimulates adhesion and functions of bone cells.^43,44^ Nanoscale roughness was also correlated with upregulation of osteogenic genes and ensured better interaction between the bone and the implant.^45^ Our results indicated that the nanostructured surfaces promoted hFOB viability, spreading, and functions. Also, nanoscale surface topography of ND surfaces induced higher ALP activity and calcium mineral synthesis. Since osseointegration of orthopedic implants was initially reliant on bone functions on the implant surfaces, enhanced hFOB viability, ALP activity, and mineralization on ND200 could contribute to osseointegration and success of the surgery.

### 3.3 Anti-Fouling Properties

**Figure 6:**
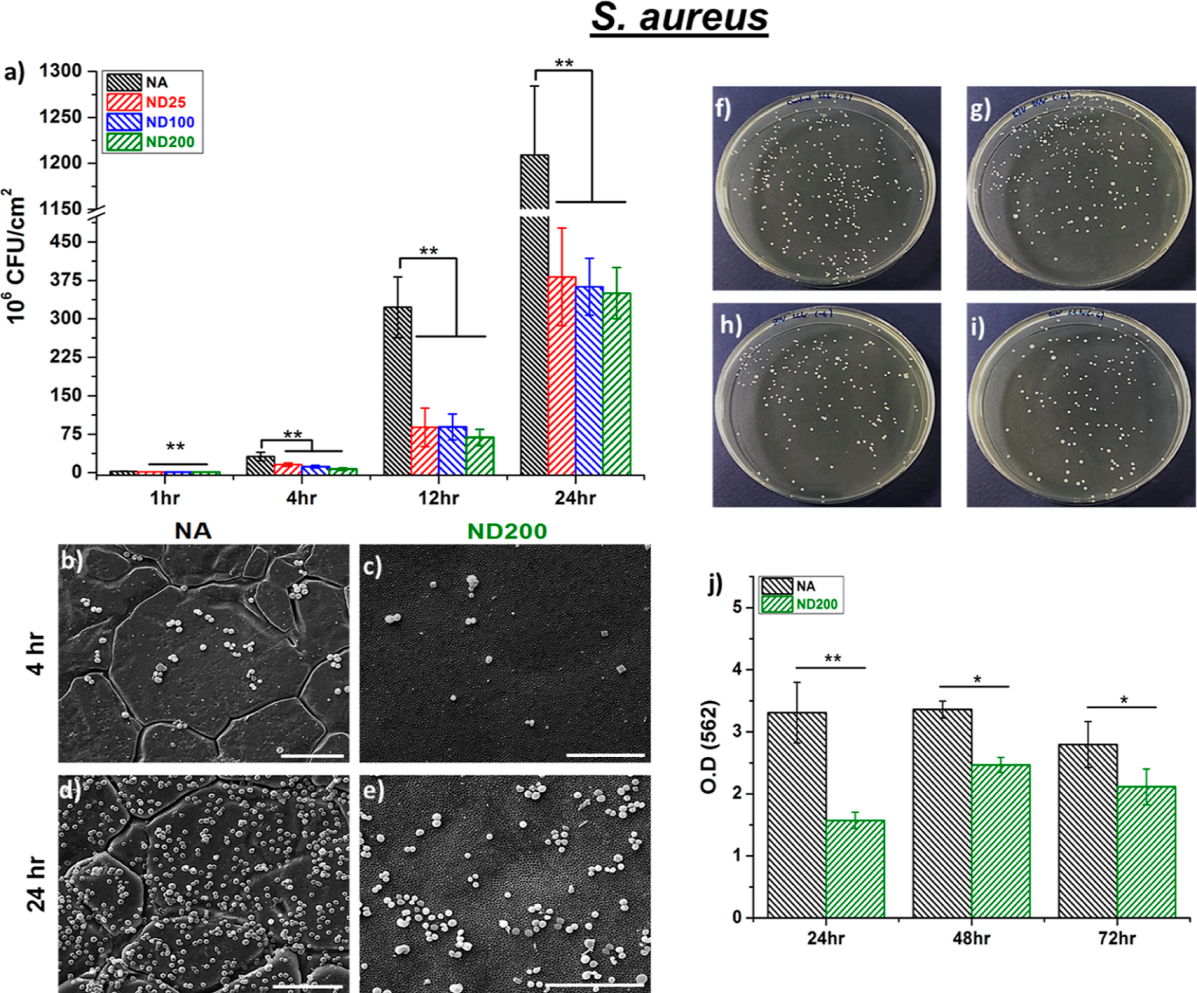
(a) *S. aureus* colony counts on NA, ND25, ND100, and ND200. SEM micrographs of *S. aureus* after (b,c) 4 h and (d,e) 24 h of culture on (b,d) NA and (c,e) ND200. Representative *S. aureus* colonies on (f) NA, (g) ND25, (h) ND100, and (i) ND200 for 24 h of culture. (j) *S. aureus* biofilm formation on NA and ND200 (*p < 0.05 and **p < 0.01).

**Figure 7:**
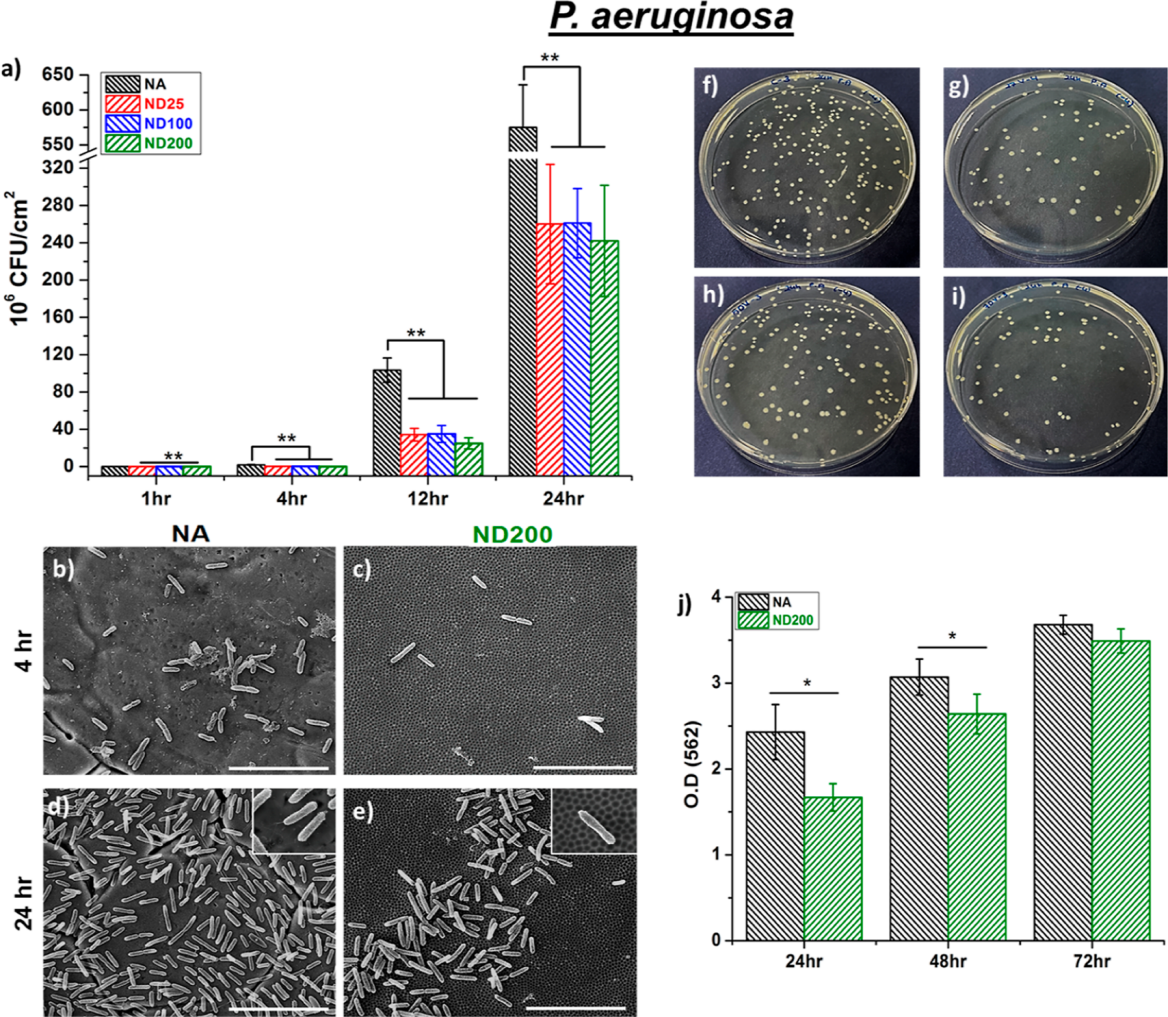
(a) *P. aeruginosa* colony counts on NA, ND25, ND100, and ND200. SEM micrographs *P. aeruginosa* after (b,c) 4 h and (d,e) 24 h of culture on (b,d) NA and (c,e) ND200. Representative *P. aeruginosa* colonies on (f) NA, (g) ND25, (h) ND100, and (i) ND200 for 24 h of culture. (j) *P. aeruginosa* biofilm formation on NA and ND200 (*p < 0.05 and **p < 0.01).

Since creating a nanostructured topography on 316LSS surfaces enhanced hFOB viability and functions, these surfaces were further investigated to assess the anti-fouling behavior of 316L SS. The adhesion and growth of *S. aureus* (Gram-positive) and *P. aeruginosa* (Gram-negative) on NA and all nanostructured surfaces were assessed by counting the colony-forming units (CFUs), as given in Figures 6 and 7. After 1 h of incubation, the quantities of adherent *S. aureus* colonies were 61, 62, and 66% less for ND25, ND100, and ND200, respectively, compared to NA (Figure 6a). At 4, 12, and 24 h of culture, *S. aureus* growth on ND200 surfaces was reduced by 78, 79, and 71%, respectively, compared to the NA surfaces. In addition, *S. aureus* growth on the ND25 surfaces was 57, 72, and 68% reduced compared to NA surfaces at 4, 12, and 24 h of culture, respectively. For the ND100 surfaces, 62, 72, and 70% reduction in CFUs was observed after 4, 12, and 24 h of incubation, respectively, compared to the NA surfaces. SEM images confirmed a similar trend that ND200 surfaces limited *S. aureus* growth compared to NA surfaces at 4 and 24 h in vitro (Figure 6b–e). Moreover, agar colony counts further confirmed that nanostructured surfaces had less *S. aureus* colonies compared to the NA surfaces (Figure 6f–i). A similar trend was also observed for Gram-negative *P. aeruginosa* growth on nanostructured surfaces. The results showed that ND25, ND100, and ND200 surfaces significantly limited adhesion of *P. aeruginosa* by 71, 70, and 77%, respectively, compared to NA after 1 h of incubation (Figure 7a). In fact, after 4, 12, and 24 h of incubation, *P. aeruginosa* colony counts were 88, 75, and 58% reduced on ND200 surfaces compared to that on the NA surface, respectively. *P. aeruginosa* CFUs on the ND25 surfaces were 86, 66, and 54% reduced compared to NA surfaces after 4, 12, and 24 h of incubation, respectively. For the ND100 surfaces, 84, 65, and 54% reduction was observed after 4, 12, and 24 h of incubation, respectively, compared to that for the NA surface. SEM micrographs in Figure 7b–e further confirm the reduced *P. aeruginosa* growth on ND200 surfaces compared to the NA surfaces at 4 and 24 h of culture in vitro. It was important to note that our experiments also showed limited biofilm growth up to 72 h on ND200 compared to NA for both bacteria strains (Figures 6j and 7j). At 24 h of incubation, the biofilms formed by *S. aureus* and *P. aeruginosa* on the ND200 surfaces were reduced by 52 and 31%, respectively, compared to that on the NA surfaces. At the end of 48 and 72 h, the biofilms formed by *S. aureus* on ND200 surfaces were 27 and 25% less, respectively, compared to that on the NA surfaces. Moreover, the biofilm formed by *P. aeruginosa* on ND200 surfaces was 15% less at the end of 48 h compared to that on NA.

**Figure 8:**
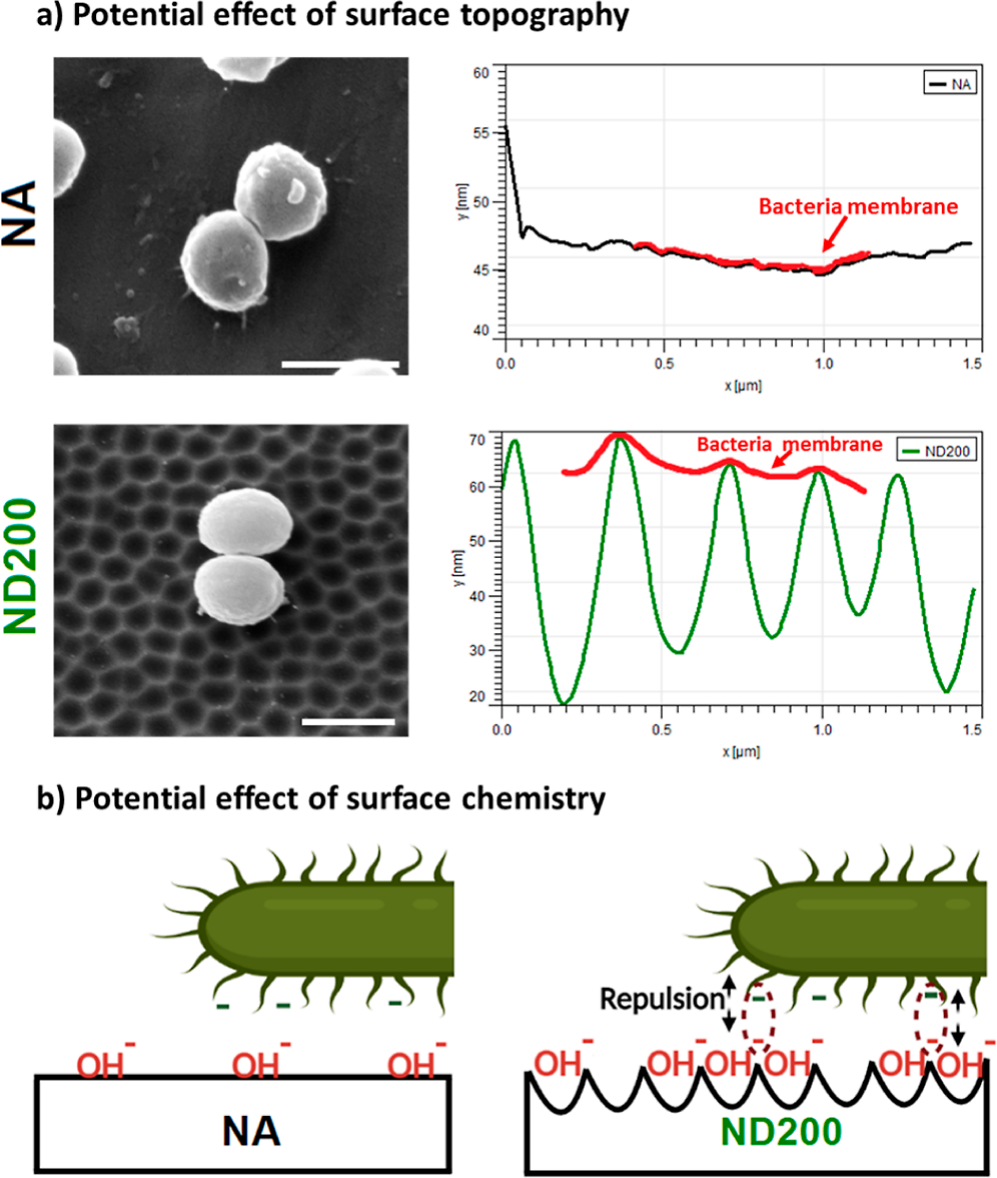
Illustration of the potential effect of (a) surface topography and (b) surface chemistry on bacterial adhesion. Topography of the ND200 surfaces led to deformation of the bacterial membranes and negative surface charge of the ND200 surfaces restricted bacterial adhesion. Scale bars are 1 μm.

The changes in surface properties, such as surface roughness, chemistry, charge, and wettability, are important to control bacterial adhesion and biofilm formation material surfaces.^46,47^ In this study, we observed that anodization process surfaces clearly played an important role in the anti-fouling properties of 316L SS.^48,49^ Though the anodization process did not change the micron scale roughness of the 316L SS surfaces, it increased their nanoscale roughness (Table 1). Our results showed that the fabrication of ND structures affected the bacteria–surface interactions and decreased bacterial attachment, growth, and biofilm formation on the 316L SS surfaces. Nanometer scale surface roughness obtained via anodization had a significant effect of limiting bacterial growth. Our results were in line with previous findings where the nanoscale surface roughness on SS was found to decrease the growth of *P. aeruginosa* and *S. aureus*.^31^ In fact, several studies showed that fabricating nanoscale surface features limited the bacterial growth despite having greater surface areas.^32,46,50^ For instance, Agbe et al. showed that the antibacterial efficiency of the anodized aluminum increased linearly with increased pore diameter and surface nanoscale roughness. They informed that 151 nm pore size effectively killed 100% of *E. coli*.^51^ The mechanism of action for anti-fouling characteristics for the nanostructured surfaces was closely linked to the nanoscale topography of the samples. It was suggested that the bacteria could attach more easily and establish more stable attachments on smooth surfaces compared to rough ones.^31^ That said, nanostructured roughness could restrain bacterial adhesion by decreasing the contact area between the bacteria and the surfaces. It was proposed that nanostructures on 316L SS led to deformation and stress on the bacterial membranes, and thus, the samples exhibited a bactericidal effect.^32^ In our study, while the nanostructured topography caused stress on the bacterial membranes, nanosmooth surfaces (i.e., NA) provided a higher contact area with bacterial membranes and set the stage for more suitable attachment, which is shown in Figure 8. It could also be speculated that anodized samples, despite having a higher total surface area, might actually have less available area for contact between the bacteria and the anodized layer due to having a dimple morphology, which had relatively deeper regions.^52^ Though bacterial cells, especially Gram-negative bacteria, could still deform, bend, and maintain their contact with the underlying surface via their appendages, the dimple morphology might limit the cell-to-surface contact area. Thus, it was possible that the area available for bacteria to attach on the anodized ND surfaces might be less than the area calculated with AFM.

In addition to the nanoscale topography, the surface chemistry of 316L SS also changed during anodization. Changes in the surface chemistry could alter the hydroxylation degree and the surface charge, which might affect bacterial adhesion and growth.^53^ Considering that bacteria possessed negatively charged surfaces due to carboxyl, amino, and phosphate functional groups on their cell membranes, they were expected to adhere more effectively onto positively charged surfaces due to electrostatic interactions.^54^ Zhu et al. showed that *E. coli* and *S. aureus* effectively adhered onto the positively charged surfaces by electrostatic attraction and, as a result, proposed negatively charged surfaces to restrict bacterial adhesion.^55^ Research conducted on anodized alumina showed that when the nanopore size on the surfaces decreased, the surface area of the samples increased, which caused enhanced repulsive forces between the surfaces and the bacteria. The increased repulsive forces, in turn, reduced the bacterial attachment onto the nanostructured surfaces.^46^ Similarly, our results showed that the ND surface topography on the anodized surfaces, independent of the size of the NDs, reduced bacterial attachment, growth, and biofilm formation compared to the NA surface lacking nanofeatures. In our study, the hydroxylation degree (OH^–^/O^2–^) increased for the ND surfaces compared to NA surfaces, which might hint at an increased negative charge on the sample surfaces. Additionally, the anodization process also increased the total surface area for the ND samples (Table 1). Since the ND surfaces had a higher surface area, which was also enriched with OH^–^, it could be speculated that the negative charge accumulated on the increased surface area of the ND samples might contribute to the anti-fouling properties (Figure 8b). Although the anti-fouling effect of negative charges on OH^–^ could be partially cancelled by the positively charged cations forming metal hydroxide on the anodized surfaces, excess OH^–^, if present, might still contribute to the decreased bacterial colonization on the samples.

To sum up, electrochemical anodization of 316L SS created a uniform array of surface features having an ND morphology. Fabrication of a nanostructured surface topography on 316L SS greatly affected the hFOB viability and bacterial growth. Nanofeatured surfaces proved to be conducive to the proliferation and mineralization of bone cells in vitro and have the potential to promote early osseointegration. Additionally, creating ND morphologies having a 200 nm feature size on 316L SS surfaces provided anti-fouling properties that decreased *S. aureus* and *P. aeruginosa* growth in vitro. To the best of our knowledge, this is the first time that the nanostructured surface topography on anodized 316L SS surfaces was demonstrated simultaneously to upregulate hFOB functions, while limiting both *S. aureus* and *P. aeruginosa* biofilm formation. According to these results, we suggest that anodization of 316L SS implant surfaces is a simple, cost-effective, and efficient method to remedy the bioinert nature and septic failure of 316L SS-based orthopedic implants, and ND morphologies having a 200 nm feature size on 316L SS were promising for bone tissue engineering applications.

## 4 Conclusions

In conclusion, we successfully obtained uniform ND structures on 316L SS surfaces using the electrochemical anodization technique. Our results suggested that ND surfaces having a 200 nm feature size enhanced hFOB viability and spreading with well-defined cytoskeletal organization. Furthermore, these nanofeatured surfaces stimulated ALP activity and promoted calcium mineral deposition. That said, ND surfaces having a 200 nm feature size showed anti-fouling activity against Gram-positive *S. aureus* and Gram-negative *P. aeruginosa* colony growth and biofilm formation. Cumulatively, fabrication of ND morphologies via anodization could remedy two major shortcomings of 316L SS implants: the bioinert nature of 316L SS and septic failure of the implants, and thus, it was a promising surface modification technique for orthopedic applications.

## References

[ref1] Bekmurzayeva A.; Duncanson W. J.; Azevedo H. S.; Kanayeva D. Surface Modification of Stainless Steel for Biomedical Applications: Revisiting a Century-Old Material. Mater. Sci. Eng., C 2018, 93, 1073–1089. 10.1016/j.msec.2018.08.049. 30274039

[ref2] Sutha S.; Kavitha K.; Karunakaran G.; Rajendran V. In-Vitro Bioactivity, Biocorrosion and Antibacterial Activity of Silicon Integrated Hydroxyapatite/Chitosan Composite Coating on 316 L Stainless Steel Implants. Mater. Sci. Eng., C 2013, 33, 4046–4054. 10.1016/j.msec.2013.05.047. 23910313

[ref3] Mohandesnezhad S.; Etminanfar M.; Mahdavi S.; Safavi M. S. Enhanced Bioactivity of 316L Stainless Steel with Deposition of Polypyrrole / Hydroxyapatite Layered Hybrid Coating : Orthopedic Applications. Surf. Interfaces 2022, 28, 101604 10.1016/j.surfin.2021.101604.

[ref4] Priyadarshini B.; Rama M.; Chetan; Vijayalakshmi U. Bioactive Coating as a Surface Modification Technique for Biocompatible Metallic Implants: A Review. J. Asian Ceram. Soc. 2019, 7, 397–406. 10.1080/21870764.2019.1669861.

[ref5] Florea D. A.; Albulet D.; Grumezescu A. M.; Andronescu E. Surface Modification – A Step Forward to Overcome the Current Challenges in Orthopedic Industry and to Obtain an Improved Osseointegration and Antimicrobial Properties. Mater. Chem. Phys. 2020, 243, 122579 10.1016/j.matchemphys.2019.122579.

[ref6] Herath I.; Davies J.; Will G.; Tran P. A.; Velic A.; Sarvghad M.; Islam M.; Paritala P. K.; Jaggessar A.; Schuetz M.; Chatterjee K.; Yarlagadda P. K. D. V. Anodization of Medical Grade Stainless Steel for Improved Corrosion Resistance and Nanostructure Formation Targeting Biomedical Applications. Electrochim. Acta 2022, 416, 140274 10.1016/j.electacta.2022.140274.

[ref7] Rasouli R.; Barhoum A.; Uludag H. A Review of Nanostructured Surfaces and Materials for Dental Implants: Surface Coating, Patterning and Functionalization for Improved Performance. Biomater. Sci. 2018, 6, 1312–1338. 10.1039/c8bm00021b. 29744496

[ref8] Davoodi E.; Zhianmanesh M.; Montazerian H.; Milani A. S.; Hoorfar M. Nano-Porous Anodic Alumina: Fundamentals and Applications in Tissue Engineering. J. Mater. Sci.: Mater. Med. 2020, 31, 60 10.1007/s10856-020-06398-2. 32642974

[ref9] Liu S.; Tian J.; Zhang W. Fabrication and Application of Nanoporous Anodic Aluminum Oxide: A Review; IOP Publishing, 2021; Vol. 32, p 222001. 10.1088/1361-6528/abe25f 33530076

[ref10] Minagar S.; Berndt C. C.; Wang J.; Ivanova E.; Wen C. A Review of the Application of Anodization for the Fabrication of Nanotubes on Metal Implant Surfaces. Acta Biomater. 2012, 8, 2875–2888. 10.1016/j.actbio.2012.04.005. 22542885

[ref11] Piszczek P.; Radtke A.; Ehlert M.; Jędrzejewski T.; Sznarkowska A.; Sadowska B.; Bartmański M.; Erdoğan Y. K.; Ercan B.; Jedrzejczyk W. Comprehensive Evaluation of the Biological Properties of Surface-Modified Titanium Alloy Implants. J. Clin. Med. 2020, 9, 342 10.3390/jcm9020342. 31991841 PMC7073575

[ref12] Sanchez A. G.; Schreiner W.; Duffó G.; Ceré S. Surface Characterization of Anodized Zirconium for Biomedical Applications. Appl. Surf. Sci. 2011, 257, 6397–6405. 10.1016/j.apsusc.2011.02.005.

[ref13] Nezhad E.; Sarraf M.; Musharavati F.; Jaber F.; Wang J. I.; Hosseini H.; Bae M.; Chowdhury S.; So M.; Sukiman H.; Liana N. Effect of Zirconia Nanotube Coating on the Hydrophilicity and Mechanochemical Behavior of Zirconium for Biomedical Applications. Surf. Interfaces 2022, 28, 101623 10.1016/j.surfin.2021.101623.

[ref14] Indira K.; Mudali U. K.; Nishimura T.; Rajendran N. A Review on TiO_2_ Nanotubes : Influence of Anodization Parameters , Formation Mechanism , Properties , Corrosion Behavior , and Biomedical Applications. J. Bio- Tribo-Corros. 2015, 1, 28 10.1007/s40735-015-0024-x.

[ref15] Mohan L.; Dennis C.; Padmapriya N.; Anandan C.; Rajendran N. Effect of Electrolyte Temperature and Anodization Time on Formation of TiO 2 Nanotubes for Biomedical Applications. Mater. Today Commun. 2020, 23, 101103 10.1016/j.mtcomm.2020.101103.

[ref16] Zhao K.; Wang S.; Lu J.; Ni C.; Wang M.; Wang S. Fabrication and Characterization of Anodic Films onto the FeCrAl Stainless Steel in Ethylene Glycol Electrolyte. Surf. Coat. Technol. 2021, 425, 127707 10.1016/j.surfcoat.2021.127707.

[ref17] Gulati K.; Santos A.; Findlay D.; Losic D. Optimizing Anodization Conditions for the Growth of Titania Nanotubes on Curved Surfaces. J. Phys. Chem. C 2015, 119, 16033–16045. 10.1021/acs.jpcc.5b03383.

[ref18] Uslu E.; Mimiroglu D.; Ercan B. Nanofeature Size and Morphology of Tantalum Oxide Surfaces Control Osteoblast Functions. ACS Appl. Bio Mater. 2021, 4, 780–794. 10.1021/acsabm.0c01354.

[ref19] Gardin C.; Ferroni L.; Erdoğan Y. K.; Zanotti F.; De Francesco F.; Trentini M.; Brunello G.; Ercan B.; Zavan B. Nanostructured Modifications of Titanium Surfaces Improve Vascular Regenerative Properties of Exosomes Derived from Mesenchymal Stem Cells: Preliminary in Vitro Results. Nanomaterials 2021, 11, 3452 10.3390/nano11123452. 34947800 PMC8707709

[ref20] Ni S.; Li C.; Ni S.; Chen T.; Webster T. J. Understanding Improved Osteoblast Behavior on Select Nanoporous Anodic Alumina. Int. J. Nanomed. 2014, 9, 3325–3334. 10.2147/IJN.S60346. PMC4099197 25045263

[ref21] Łyczkowska-Widłak E.; Lochyński P.; Nawrat G. Electrochemical Polishing of Austenitic Stainless Steels. Materials 2020, 13, 2557 10.3390/ma13112557. 32512733 PMC7321480

[ref22] Benčina M.; Junkar I.; Vesel A.; Mozetič M.; Iglič A. Nanoporous Stainless Steel Materials for Body Implants—Review of Synthesizing Procedures. Nanomaterials 2022, 12, 2924 10.3390/nano12172924. 36079962 PMC9457931

[ref23] Latifi A.; Imani M.; Khorasani M. T.; Joupari M. D. Electrochemical and Chemical Methods for Improving Surface Characteristics of 316L Stainless Steel for Biomedical Applications. Surf. Coat. Technol. 2013, 221, 1–12. 10.1016/j.surfcoat.2013.01.020.

[ref24] Dhawan U.; Pan H. A.; Shie M. J.; Chu Y. H.; Huang G. S.; Chen P. C.; Chen W. L. The Spatiotemporal Control of Osteoblast Cell Growth, Behavior, and Function Dictated by Nanostructured Stainless Steel Artificial Microenvironments. Nanoscale Res. Lett. 2017, 12, 86 10.1186/s11671-016-1810-1. 28168610 PMC5293702

[ref25] González A. S.; Riego A.; Vega V.; García J.; Galié S.; Gutiérrez del Río I.; Martínez de Yuso M. V.; Villar C. J.; Lombó F.; De la Prida V. M. Functional Antimicrobial Surface Coatings Deposited onto Nanostructured 316l Food-grade Stainless Steel. Nanomaterials 2021, 11, 1055 10.3390/nano11041055. 33924070 PMC8074267

[ref26] Rodriguez-Contreras A.; Guadarrama Bello D.; Flynn S.; Variola F.; Wuest J. D.; Nanci A. Chemical Nanocavitation of Surfaces to Enhance the Utility of Stainless Steel as a Medical Material. Colloids Surf., B 2018, 161, 677–687. 10.1016/j.colsurfb.2017.11.051. 29175762

[ref27] Hsu H. J.; Wu C. Y.; Huang B. H.; Tsai C. H.; Saito T.; Ou K. L.; Chuo Y. C.; Lin K. L.; Peng P. W. Surface Characteristics and Cell Adhesion Behaviors of the Anodized Biomedical Stainless Steel. Appl. Sci. 2020, 10, 6275 10.3390/APP10186275.

[ref28] Cho Y. C.; Hung W. C.; Lan W. C.; Saito T.; Huang B. H.; Lee C. H.; Tsai H. Y.; Huang M. S.; Ou K. L. Anodized Biomedical Stainless-Steel Mini-Implant for Rapid Recovery in a Rabbit Model. Metals 2021, 11, 1575 10.3390/met11101575.

[ref29] Resnik M.; Benčina M.; Levičnik E.; Rawat N.; Iglič A.; Junkar I. Strategies for Improving Antimicrobial Properties of Stainless Steel. Materials 2020, 13, 2944 10.3390/ma13132944. 32630130 PMC7372344

[ref30] Chopra D.; Gulati K.; Ivanovski S. Understanding and Optimizing the Antibacterial Functions of Anodized Nano-Engineered Titanium Implants. Acta Biomater. 2021, 127, 80–101. 10.1016/j.actbio.2021.03.027. 33744499

[ref31] Wu S.; Altenried S.; Zogg A.; Zuber F.; Maniura-Weber K.; Ren Q. Role of the Surface Nanoscale Roughness of Stainless Steel on Bacterial Adhesion and Microcolony Formation. ACS Omega 2018, 3, 6456–6464. 10.1021/acsomega.8b00769. 30023948 PMC6045408

[ref32] Jang Y.; Choi W. T.; Johnson C. T.; García A. J.; Singh P. M.; Breedveld V.; Hess D. W.; Champion J. A. Inhibition of Bacterial Adhesion on Nanotextured Stainless Steel 316L by Electrochemical Etching. ACS Biomater. Sci. Eng. 2018, 4, 90–97. 10.1021/acsbiomaterials.7b00544. 29333490 PMC5761049

[ref33] Domínguez-Jaimes L. P.; Arenas Vara M. Á. A.; Cedillo-González E. I.; Ruiz Valdés J. J. R.; De Damborenea J. J.; Conde Del Campo A.; Rodríguez-Varela F. J.; Alonso-Lemus I. L.; Hernández-López J. M. Corrosion Resistance of Anodic Layers Grown on 304L Stainless Steel at Different Anodizing Times and Stirring Speeds. Coatings 2019, 9, 706 10.3390/coatings9110706.

[ref34] Habibzadeh S.; Li L.; Shum-Tim D.; Davis E. C.; Omanovic S. Electrochemical Polishing as a 316L Stainless Steel Surface Treatment Method: Towards the Improvement of Biocompatibility. Corros. Sci. 2014, 87, 89–100. 10.1016/j.corsci.2014.06.010.

[ref35] Zhao J.; Zhai Z.; Sun D.; Yang C.; Zhang X.; Huang N.; Jiang X.; Yang K. Antibacterial Durability and Biocompatibility of Antibacterial-Passivated 316L Stainless Steel in Simulated Physiological Environment. Mater. Sci. Eng., C 2019, 100, 396–410. 10.1016/j.msec.2019.03.021. 30948076

[ref36] Biesinger M. C.; Payne B. P.; Grosvenor A. P.; Lau L. W. M.; Gerson A. R.; Smart R. Resolving Surface Chemical States in XPS Analysis of First Row Transition Metals , Oxides and Hydroxides : Cr , Mn , Fe , Co and Ni. Appl. Surf. Sci. 2011, 257, 2717–2730. 10.1016/j.apsusc.2010.10.051.

[ref37] Zhang B.; Ni H.; Chen R.; Zhan W.; Zhang C.; Lei R.; Zha Y. A Two-Step Anodic Method to Fabricate Self-Organised Nanopore Arrays on Stainless Steel. Appl. Surf. Sci. 2015, 351, 1161–1168. 10.1016/j.apsusc.2015.06.083.

[ref38] Wang Z.; Di-Franco F.; Seyeux A.; Zanna S.; Maurice V.; Marcus P. Passivation-Induced Physicochemical Alterations of the Native Surface Oxide Film on 316L Austenitic Stainless Steel. J. Electrochem. Soc. 2019, 166, C3376 10.1149/2.0321911jes.

[ref39] Nagai A.; Suzuki Y.; Tsutsumi Y.; Nozaki K.; Wada N.; Katayama K.; Hanawa T.; Yamashita K. Anodic oxidation of a Co-Ni-Cr-Mo alloy and its inhibitory effect on platelet activation. J. Biomed. Mater. Res., Part B 2013, 102, 659–666. 10.1002/jbm.b.33044. 24843884

[ref40] Yong X.; Xiao N.; Shen H.; Song Y. Responses of the Corroded Surface Layer of Austenitic Stainless Steel to Different Corrosive Conditions under Cavitation. Mater. Sci. Eng. A 2016, 671, 118–126. 10.1016/j.msea.2016.06.019.

[ref41] Beltrán-Partida E.; Valdéz-Salas B.; Moreno-Ulloa A.; Escamilla A.; Curiel M. A.; Rosales-Ibáñez R.; Villarreal F.; Bastidas D. M.; Bastidas J. M. Improved in vitro angiogenic behavior on anodized titanium dioxide nanotubes. J. Nanobiotechnol. 2017, 15, 10 10.1186/s12951-017-0247-8. PMC5282661 28143540

[ref42] Pan H. A.; Liang J. Y.; Hung Y. C.; Lee C. H.; Chiou J. C.; Huang G. S. The Spatial and Temporal Control of Cell Migration by Nanoporous Surfaces through the Regulation of ERK and Integrins in Fibroblasts. Biomaterials 2013, 34, 841–853. 10.1016/j.biomaterials.2012.09.078. 23131534

[ref43] Luo J.; Walker M.; Xiao Y.; Donnelly H.; Dalby M. J.; Salmeron-Sanchez M. The Influence of Nanotopography on Cell Behaviour through Interactions with the Extracellular Matrix—A Review. Bioact. Mater. 2022, 15, 145–159. 10.1016/j.bioactmat.2021.11.024. 35386337 PMC8940943

[ref44] Le X.; Poinern G.; Ali J.; Berry N.; Fawcett C. M.; Fawcett D. Engineering a Biocompatible Scaffold with Either Micrometre or Nanometre Scale Surface Topography for Promoting Protein Adsorption and Cellular Response. Int. J. Biomater. 2013, 2013, 782549 10.1155/2013/782549. 23533416 PMC3600176

[ref45] Ni S.; Zhai D.; Huan Z.; Zhang T.; Chang J.; Wu C. Nanosized Concave Pit/Convex Dot Microarray for Immunomodulatory Osteogenesis and Angiogenesis. Nanoscale 2020, 12, 16474–16488. 10.1039/d0nr03886e. 32743625

[ref46] Feng G.; Cheng Y.; Wang S. Y.; Hsu L. C.; Feliz Y.; Borca-Tasciuc D. A.; Worobo R. W.; Moraru C. I. Alumina Surfaces with Nanoscale Topography Reduce Attachment and Biofilm Formation by Escherichia Coli and Listeria Spp. Biofouling 2014, 30, 1253–1268. 10.1080/08927014.2014.976561. 25427545

[ref47] Cheng Y.; Feng G.; Moraru C. I. Micro-and Nanotopography Sensitive Bacterial Attachment Mechanisms: A Review. Front. Microbiol. 2019, 10, 1–17. 10.3389/fmicb.2019.00191. 30846973 PMC6393346

[ref48] Liu L.; Ercan B.; Sun L.; Ziemer K. S.; Webster T. J. Understanding the Role of Polymer Surface Nanoscale Topography on Inhibiting Bacteria Adhesion and Growth. ACS Biomater. Sci. Eng. 2016, 2, 122–130. 10.1021/acsbiomaterials.5b00431. 33418649

[ref49] Ercan B.; Taylor E.; Alpaslan E.; Webster T. J. Diameter of Titanium Nanotubes Influences Anti-Bacterial Efficacy. Nanotechnology 2011, 22, 295102 10.1088/0957-4484/22/29/295102. 21673387

[ref50] Singh A. V.; Vyas V.; Patil R.; Sharma V.; Scopelliti P. E.; Bongiorno G.; Podestà A.; Lenardi C.; Gade W. N.; Milani P. Quantitative Characterization of the Influence of the Nanoscale Morphology of Nanostructured Surfaces on Bacterial Adhesion and Biofilm Formation. PLoS One 2011, 6, 25029 10.1371/journal.pone.0025029. PMC3180288 21966403

[ref51] Agbe H.; Sarkar D. K.; Chen X. G. Anodized Aluminum Surface with Topography-Mediated Antibacterial Properties. ACS Biomater. Sci. Eng. 2022, 8, 1087–1095. 10.1021/acsbiomaterials.1c01485. 35195412

[ref52] Feng G.; Cheng Y.; Wang S. Y.; Borca-Tasciuc D. A.; Worobo R. W.; Moraru C. I. Bacterial Attachment and Biofilm Formation on Surfaces Are Reduced by Small-Diameter Nanoscale Pores: How Small Is Small Enough?. npj Biofilms Microbiomes 2015, 1, 15022 10.1038/npjbiofilms.2015.22. 28721236 PMC5515209

[ref53] Li S.; Liu Y.; Zheng Z.; Liu X.; Huang H.; Han Z.; Ren L. Biomimetic Robust Superhydrophobic Stainless-Steel Surfaces with Antimicrobial Activity and Molecular Dynamics Simulation. Chem. Eng. J. 2019, 372, 852–861. 10.1016/j.cej.2019.04.200.

[ref54] Zheng S.; Bawazir M.; Dhall A.; Kim H. E.; He L.; Heo J.; Hwang G. Implication of Surface Properties, Bacterial Motility, and Hydrodynamic Conditions on Bacterial Surface Sensing and Their Initial Adhesion. Front. Bioeng. Biotechnol. 2021, 9, 643722 10.3389/fbioe.2021.643722. 33644027 PMC7907602

[ref55] Zhu X.; Jańczewski D.; Guo S.; Lee S. S. C.; Parra Velandia F. J.; Teo S. L. M.; He T.; Puniredd S. R.; Vancso G. J. Polyion Multilayers with Precise Surface Charge Control for Antifouling. ACS Appl. Mater. Interfaces 2015, 7, 852–861. 10.1021/am507371a. 25485625

